# Side effects of hydroxyurea in patients with Thalassemia major and thalassemia intermedia and sickle cell anemia

**Published:** 2014-07-20

**Authors:** A Ghasemi, B Keikhaei, R Ghodsi

**Affiliations:** 1 Assistant professor of pediatric hematology and oncology, faculty of medicine , Mashhad University of Medical Sciences, Mashhad,Iran.; 2 Associate professor of pediatric hematology and oncology, Jondishapour University of Medical Sciences, Ahvaz, (research center for thalassemia and hemoglobinopathy).; 3 Department of Medicinal Chemistry, School of Pharmacy, Mashhad University of Medical Sciences, Mashhad/Iran

**Keywords:** Side effects, hydroxyurea, β-Thalasemia, Sickle cell anemia

## Abstract

**Background:**

Sickle hemoglobin is the most common abnormal hemoglobin in the United States. Hemoglobin S arises as a result of a single amino acid substitution (glutamic acid to valin at position 6 of the β-globine chain).

The presence of fetal hemoglobin (HbF) plays a relatively protective role since a significant amount of HbF interferes with HbS polymerization, the pathogenesis mechanism of the vaso-occlusive symptoms that are the major contributor of the morbidity and mortality of this condition

Thalassemia major and thalassemia intermedia have no specific molecular correlate but encompass a wide spectrum of clinical and laboratory abnormalities.

Hydroxyurea (HU), an s-phase-specific and non-DNA-hypomethylating chemotherapeutic agents is capable of inducing HbF synthesis.

**Materials and Methods:**

This study was done on 56 patients, 28 patients with sickle cell anemia (SCA) and 28 patients with intermediate or major β-thalassemia.

Start dose of HU was 10 mg/kg per day and increased by 5 mg/kg per day every 4-6 weeks until toxicity or according to clinical response.

**Results:**

The side effects were dermatologic in 39.28%, neurologic 23.2%, gastrointestinal 17.5% and hematologic 10.71% of patients. the statistical analysis didn't show significant relationship between variables such as history of blood transfusion, duration of HU treatment, age of start HU, age of diagnosis, dose of HU and ethnic with occurrence of HU adverse effects.

**Conclusion:**

The HU therapy in our patients tolerated well and side effects were minor to moderate, benign and transient.

## Introduction

Sickle hemoglobin is the most common abnormal hemoglobin in the United States. Hemoglobin S arises as a result of a single amino acid substitution (glutamic acid to valin at position 6 of the β-globine chain) (1 )

In patients with sickle cell anemia, the presence of fetal hemoglobin (HbF) in infancy plays a relatively protective role since a significant amount of HbF interferes with HbS polymerization, the pathogenesis mechanism of the vaso-occlusive symptoms that are the major contributor of the morbidity and mortality of this condition (2).

Thalassemia major and thalassemia intermedia have no specific molecular correlate but encompass a wide spectrum of clinical and laboratory abnormalities (3).

Patients referred to as having thalassemia major are usually those who come to medical attention in the first year of life and subsequently require regular transfusions to survive. Those who present later or who seldom need transfusions are said to have thalassemia intermedia (4).

Hydroxycarbamide, also known as hydroxyurea, an S-phase specific and non-DNA-hypomethylating chemotherapeutic agents is capable of inducing HbF synthesis, the effect of hydroxyurea and other antimetabolits on HbF synthesis is mainly mediated by their cytotoxic properties (5).

Hydroxyurea produces fetal hemoglobin production via a reactivation of Υgenes as a result of some unknown molecular mechanisms (6).

The commonest side-effect is dose-dependent myelosuppression: Although this is usually transient (7).

Cutaneous side-effect includes nail hyper pigmentation, which is common and increased skin pigmentation, especially on the palms and soles. Hydroxyurea is associated with development of leg ulcers in myeloprolifrative diseased and some studies have rates of up to 30% in SCD patients on hydroxyurea (8,9).

Nausea, rash and other gasterointestinal upsets have been described with hydroxyurea (10).

Hydroxyurea is renally excreted and small increases in creatinine are sometimes seen on treatment.

## Materials and Methods

This was a cross-sectional study, sampling was simple random, data gathered with a questionnaire. We included 56 patients, 28 patients with sickle cell anemia and 28 patients with intermediate or major β-thalassemia.

Exclusion criteria for all patients were creatinine level more than twice the upper limit of normal for age or greater than1.5 mg/dl and active liver disease. 

These patients visited monthly by pediatric specialist and pediatric hematologist-oncologist and evaluated about hydroxyurea complicatious. Laboratory tests include CBC (complex blood count) and liver enzymes performed monthly. Start dose of HU was 10 mg/kg per day and increased by 5 mg/kg per day every 4-6 weeks until toxicity or according to clinical response or total dose 35mg/kg/day.

The variables shush as gender, age, ethnic, age of diagnosis, age of start HU, history of blood transfusion, duration of HU treatment, dose of HU, recorded and compared correlation with side effects such as dermatologic, neurologic, hematologic, hepatic and others.

**Statistical analysis**

The data analyzed with software SPSS version 16 and significant level was P-Value < 0.05.

## Results

This study was to evaluate the side effects of HU treatment in 56 patients, 24 male (42.9%) and 32 female (57.1%) our report investigate results in two group of patients 

a) Twenty-eight Patients with sickle cell anemia treated with HU.

b) Twenty-eight Patients with major and β-thalassemia.

 Ethnic of our patients were 37 Arabian (66.1%) and 19 non-Arabian (33.9%) 

They had a median age of 17.5±8.55 years (range: 4-52 years).

Forty-five patients (80.4%) had history of transfusion, 29 of transfused patients suffered complication.

Side effects of HU have been recorded in 37 (66.1%) patients (Table I).

The median age of patient diagnosis was 4.25±6.32 years and 14±10.4 years in start of HU.

The duration of HU treatment was 2.63±2.28 years.

The mean dose of HU in our patients was 15.54±3.02 mg/kg per day

The most common side effect was dermatologic which occurred in 22 ( 39.28% ) of them. The major cutaneous side effects were hair loss 33.9% (19 patients), hyper pigmentation 10.7% (6 patients), skin rash 3.57% (2 patients) and nail hyper pigmentation 1.7%.

The gastrointestinal adverse effects of HU recorded in 10 ( 17.5% ) of our patients and included nausea and vomiting 5.36%(3 patients), abdominal pain 8.9%(5 patients), anorexia 3.57%(2 patients) and constipation 1.7%(1 patient).

The prevalence of neurologic side effects was 23.2% (13 patients) included headache (16%), vertigo (3.57%), drowsiness (1.7%) and seizure (1.7%).

Other adverse effects included increase(2 times over upper limit of normal) in AST-ALT 7.4% (4 patients), weight gain 3.57% (2 patients), skin ulcer 1.7% (1 patient), and edema 1.7% (1 patient). 

Hematologic side effects included decrease in PLT 3.57% (2 patients), neutropenia 3.57% (2 patients) and decrease in Hb level 3.57% (2patients).

**Table I:** Side effects of hydoxyurea

**Side effects**		**Patients (%)**
**Neurologic 13** **(** **23.2%** **)**	Headache	13(16)
	Vertigo	2 (3.57)
	Drowsiness	1(1.7)
	Seizure	1(1.7)
**Dermatologic 22(39.28%)**	Hair Loss	19 (33.9)
	Hyper pigmentation	6 (10.7)
	Skin ulcer	1(1.7)
	Skin rash	2(3/57)
	Nail hyper pigmentation	1(1.7)
**Gastrointestinal 10(17.5%)**	Abdominal Pain	5(8.9)
	Nausea and vomiting	3(5.3)
	Constipation	1(1.7)
	Anorexia	2 (3.57)
**Hematologic** **6(10.7)**	Decreased PLT	2 (3.57)
	Decreased HB	2 (3.57)
	Neutropenia	2 (3.57)
**Others**	Increased AST,ALT	4(7.4)
	Weight gain	2(3.57)
	Edema	1(1.7)

## Discussion

The most common side effects were hair loss and headache in 33.9% and 16% of patients respectly.

Hyperpigmentation has been recorded in 10.7% patients.

 The side effects were dermatologic in 39.28 %( 23), neurologic 23.2 %(13), gastrointestinal 17.5%(10) and hematologic 10.71% (6) of patients.

 Chick and coworkers (2006) didn’t encounter leucopenia or abnormality in renal and liver functions. One patient had reduction in the nucleated red blood cell in peripheral blood. (7)

In Benedict study recorded that the most common side effect was dermatologic and hyper pigmentation was most common dermatologic side effect (8).

In our study, the most common side effect was dermatologic in39% patients.

Zargari and co workers retorted, the cutaneous effects of HU have been described for patients with sickle cell anemia, myeloprolifrative disorders, and psoriasis, there are no reported of cutaneous adverse effects from HU when used for patients with intermediate thalassemia (9). Ferguson and coworkers described that HU improved clinical outcomes in patients with sickle cell anemia (10).

Karimi and coworkers reported that dermatologic side effects were most commonly seen, followed by neurologic and gastrointestinal adverse effects. There were not any reports of hematologic toxicity or any signs of bone marrow suppression during HU treatment (11).

 Statistical analysis showed a positive correlation between advancing age and the presence of adverse effects during HU treatment, but there were not any significant relations among gender, HU dose and duration of HU treatment and the presence of adverse effects (P>005). 

Hope and coworkers suggests that hydroxyurea is safe and effective in sickle cell disease (12).

In our study, the statistical analysis didn't show significant relationship between variables such as history of blood transfusion, duration of HU treatment, age of start HU, age of diagnosis, dose of HU and ethnic with occurrence of HU adverse effects.

## Conclusion

Based on the evidence of several studies, HU plays an important role in the management of a number of complication of SCD and decrease transfusion number of thalassemic patients and saves them from side effects of blood transfusions.

The HU therapy in our patients tolerated well and side effects were minor to moderate, benign and transient. We recommend, to starting low dose of HU (10 mg/kg per day) and increase dose slowly in pediatric and adult patients with SCA and thalassemia can be tolerated well without serious side effects. 

## Conflict of interest

The authors have no conflict of interest.
